# Non-cardioembolic risk factors in atrial fibrillation-associated ischemic stroke

**DOI:** 10.1371/journal.pone.0201062

**Published:** 2018-07-20

**Authors:** Pil-Sung Yang, Hui-Nam Pak, Dong-Hyuk Park, Joonsang Yoo, Tae-Hoon Kim, Jae-Sun Uhm, Young Dae Kim, Hyo Suk Nam, Boyoung Joung, Moon-Hyoung Lee, Ji Hoe Heo

**Affiliations:** 1 Division of Cardiology, Department of Internal Medicine, Yonsei University Health System, Seoul, Republic of Korea; 2 Department of Neurology, Yonsei University Health System, Seoul, Republic of Korea; University of Palermo, ITALY

## Abstract

**Introduction:**

Cardioembolic (CE) risks is usually considered as the main mechanism of ischemic stroke in non-valvular atrial fibrillation (NVAF) patients. However, a substantial number of ischemic strokes in NVAF patients are related to non-CE mechanisms. The aim of this study was to investigate the non-CE risk factors in ischemic stroke patients had NVAF.

**Methods:**

We included 401 patients (65.6% male, 68.6 ± 9.6 years old) who had been hospitalized due to ischemic stroke and had a known or newly diagnosed NVAF. The CE (intracardiac thrombus, dense spontaneous echo contrast, or low left atrial appendage flow velocity) and non-CE (complex aortic plaque, significant carotid stenosis, or intracranial arterial stenosis) risk factors were investigated at the time of the index stroke.

**Results:**

The number of CE and non-CE risk factors increased with increasing CHA_2_DS_2_-VASc scores (p for trends < 0.001). The presence of CE risk factors was independently associated with persistent atrial fibrillation (p < 0.001), body mass index (p = 0.003), heart failure (p = 0.003), and left atrial volume index (p < 0.001). In contrast, the presence of non-CE risk factors was independently associated with age (p < 0.001), hypertension (p = 0.049), diabetes (p = 0.030), and coronary artery calcium score (CACS; p < 0.001). CACS had the added value in predicting non-CE risk factors of ischemic stroke regardless of the CHA_2_DS_2_-VASc risk category (p < 0.001).

**Conclusion:**

Non-CE risk factors in ischemic stroke patients with NVAF are associated with high CHA_2_DS_2_-VASc score and CACS. Atherosclerotic non-CE risk factors should be considered as potential mechanisms of stroke even in patients with AF-associated ischemic stroke.

## Introduction

Non-valvular atrial fibrillation (NVAF) is the most common arrhythmic cause of ischemic stroke, and anticoagulation is highly effective for stroke prevention in NVAF patients. However, anticoagulation also can increase the risk of bleeding [1]. Therefore, risk stratification schemes such as the CHADS_2_ and CHA_2_DS_2_-VASc scores have been developed to identify patients eligible for anticoagulation [2, 3]. Recent European Society of Cardiology (ESC) guidelines for atrial fibrillation (AF) management in 2016 determined that anticoagulation or antiplatelet therapy was contraindicated in patients with CHA_2_DS_2_-VASc scores of 0, but anticoagulation mono-therapy has been generally accepted for high risk patients [3]. Loss of mechanical function of the atrium has been considered the main mechanism of ischemic stroke-associated cardioembolic (CE) risks, such as intracardiac thrombus, dense spontaneous echo contrast (SEC), or low left atrial appendage (LAA) flow velocity [4]. However, a substantial number of ischemic strokes in NVAF patients can be caused by atherosclerotic non-CE risks, such as complex aortic plaque [5], significant carotid stenosis [6], or intra-cranial arterial stenosis [7], especially in patients with high CHA_2_DS_2_-VASc scores. However, few data are available regarding the relationship between the overall burden of non-CE risk factors and CHA_2_DS_2_-VASc scores in NVAF patients.

Therefore, we investigated the burden of CE and non-CE risk factors of ischemic stroke according to the CHA_2_DS_2_-VASc score in patients who had been hospitalized due to ischemic stroke and had a history of AF or detection of AF on monitoring after stroke. We also investigated the clinical characteristics of patients with non-CE risk factors to identify clinical markers that can be used to identify patients with non-CE risk factors in addition to risk stratification schemes.

## Methods

### Study population

The study protocol adhered to the Declaration of Helsinki and was approved by the Institutional Review Board (IRB) of Yonsei University Health System. The IRB waived the requirement to obtain informed consent. This study was a retrospective review of medical records and examinations. From the medical records of Severance Hospital, Yonsei University Health System between January 2006 and May 2015, we retrospectively enrolled 401 non-consecutive patients (65.6% male, 68.6±9.6 years old) who were hospitalized due to AF–associated ischemic stroke and underwent all of the following examinations to establish the cause of stroke: trans-thoracic echocardiography (TTE), trans-esophageal echocardiography (TEE), cardiac computed tomography (CT), carotid duplex sonography, and brain CT angiography (CTA) and/or magnetic resonance angiography (MRA). AF–associated ischemic stroke was defined as an ischemic stroke and history of AF or detection of AF on monitoring after stroke. The study`s exclusion criteria were as follows: 1) those with valvular AF (moderate to severe mitral stenosis, any mechanical or bioprosthetic heart valve, or mitral valve repair), 2) those with stroke due to infective endocarditis or cardiac myxoma, 3) and those with an atrial septal defect or large patent foramen ovale.

### Definition of CE and non-CE risk factors of ischemic stroke

**Fig 1:**
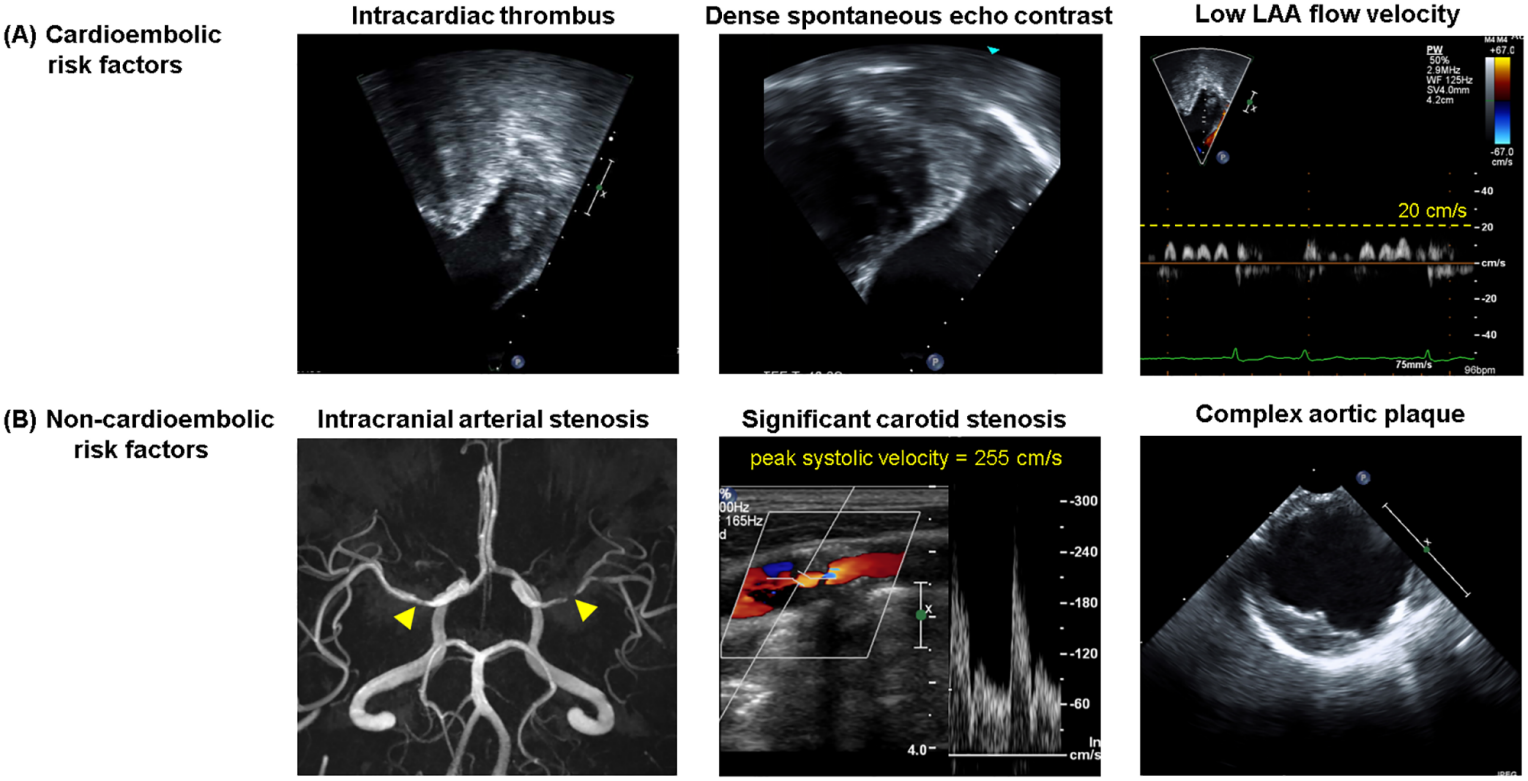
Representative images of (A) cardioembolic (CE) and (B) non-CE risk factors in patients with non-valvular atrial fibrillation. In intracranial arterial stenosis (B), the yellow arrow indicates the atherosclerotic stenosis lesion on magnetic resonance angiography. LAA = left atrial appendage.

CE risk factors of ischemic stroke were defined as intracardiac thrombus, intracardiac dense SEC, and low LAA flow velocity (Fig 1A) [4]. To investigate these CE risk factors, we reviewed the reports and digitally stored images from TTE and TEE in all patients while blinded to the clinical data and risk scores. Intracardiac thrombus was defined as a discrete mass seen in multiple windows that was separate from the endocardium and pectinate muscles within the left ventricle (LV), left atrium (LA), or LAA in TTE or TEE images. SEC in the LA or LAA was classified as none, faint, or dense from TEE images with optimal gain settings [8, 9]. For assessment of LAA flow velocity, five consecutive pulsed-wave Doppler outflow velocity signals during diastole were measured by TEE at 1 cm below the orifice of the LAA over at least three cardiac cycles and averaged. Low LAA flow velocity was defined as ≤20 cm/s [8, 9].

Non-CE risk factors of ischemic stroke were defined as atherosclerotic intracranial arterial stenosis [7, 10], significant carotid stenosis [6, 11], and complex aortic plaque (Fig 1B) [5]. To investigate these non-CE risk factors, we reviewed the reports and digitally stored images from brain CTA/MRA, carotid duplex sonography, and TEE in all patients while blinded to clinical data and risk scores. Atherosclerotic intracranial arterial stenosis was investigated using the results of brain CTA/MRA reported by experienced neuroradiologists. Steno-occlusive lesions in a symptomatic intracranial artery leading to infarcted areas were excluded from the atherosclerotic intracranial arterial stenosis to avoid erroneous inclusion of steno-occlusive lesions due to embolism from the heart. In patients who underwent CTA and MRA, the results of CTA were used for analysis. Significant carotid stenosis was defined as ≥50% diameter stenosis, peak systolic velocity of the right or left common or internal carotid artery ≥150 cm/s, or occlusion in carotid duplex sonography [11]. Aortic plaque was defined as protrusions different in appearance and echogenicity from the adjacent intimal surface of the aorta on TEE. Complex aortic plaque was defined as large (≥4 mm in thickness measured in the horizontal plane), or as having ulcerations or mobile components [5].

### Clinical variables and CHA_2_DS_2_-VASc score

Demographic characteristics and clinical variables of each patient were obtained via review of electronic medical records. Clinical variables of interest included history of hypertension, diabetes mellitus, heart failure, previous ischemic stroke, or transient ischemic attack (TIA), myocardial infarction (MI), and peripheral artery disease (PAD). AF was classified as paroxysmal or persistent from serial 12-lead electrocardiograms and 24 hour Holter monitoring according to definitions from the American Heart Association (AHA) and the European Society of Cardiology (ESC) guidelines [2, 3]. The CHA_2_DS_2_-VASc scores (range, 0–9) were retrospectively calculated according to definitions in the AHA and the ESC guidelines [2, 3]. Index ischemic stroke was not considered in the calculation. The definition of vascular disease used for the CHA_2_DS_2_-VASc score was a previous MI, PAD, or the existence of a complex aortic plaque on TEE [12]. However, only complex aortic plaque found before index ischemic stroke was used for CHA_2_DS_2_-VASc score calculation to evaluate the patient based on the time before the index ischemic stroke occurred.

### Coronary artery calcium score

All patients underwent cardiac CT to evaluate concomitant coronary artery disease and coronary artery calcium score (CACS). Patients were scanned with a ≥ 64-section CT scanner. Coronary artery calcium was identified as a high-attenuation area in the coronary artery whose attenuation exceeded the threshold of 130 Hounsfield units in a minimum of 3 contiguous pixels. CACS was calculated according to the Agatston method [13].

### Statistical analysis

Continuous variables were expressed as mean ± standard deviation and categorical variables were expressed as counts and percentages. A student’s t test for continuous variables or Fisher’s exact test for categorical variables was used to determine the significance of differences in variables between two groups. P values for trends were calculated using the Cochran-Armitage test. Multivariate logistic regression analysis with pre-specified model was used to identify the independent predictors of the presence of CE or non-CE stroke risk factors in patients with NVAF. All models were based upon age, sex, and clinical variables that were statistically significant after univariate analysis. Models with interactions were also evaluated, but no significant interactions were found. A p-value less than 0.05 was considered statistically significant. All statistical analyses were performed using SPSS version 23.0 (IBM SPSS Inc., Chicago, IL, USA).

## Results

### Clinical characteristics associated with CE or non-CE risk factors

Table 1 shows the characteristics of the 401 NVAF patients in the study (age: 68.6±9.6 years old, 65.6% male, 47.5% persistent AF), and compares patients with CE or non-CE risk factors to those without. The overall mean CHA_2_DS_2_-VASc scores was 3.14±1.67 (Index ischemic stroke was not considered in the calculation). CE risk factors were found in 142 (35.4%) patients, and non-CE risk factors were found in 183 (45.6%) patients. Both CE and non-CE risk factors were common in elderly patients with a high AF burden (persistent AF) and larger LA size (Table 1). CE risk factors were related to larger body mass index (BMI) and heart failure, while non-CE risk factors were associated with hypertension, diabetes, low estimated glomerular filtration rate (eGFR), previous MI or PAD, and high CACS.

**Table 1:** Patient characteristics.

Characteristic	All NVAF patients (n = 401)	With CE risk factors ^a^ (n = 142)	Without CE risk factors (n = 259)	p value	With non-CE risk factors ^b^ (n = 183)	Without non-CE risk factors (n = 218)	p value
Age (years)	68.6±9.6	70.16±8.96	67.69±9.86	**0.014**	71.8±8.2	65.8±9.9	**<0.001**
Sex (male)	263 (65.6%)	94 (66.2%)	169 (65.3%)	0.913	121 (66.1%)	142 (65.1%)	0.916
Persistent AF	191 (47.5%)	102 (71.8%)	88 (34.1%)	**<0.001**	98 (53.6%)	92 (42.4%)	**0.027**
Body mass index (kg/m^2^)	23.9±3.8	24.43±3.39	23.58±3.94	**0.030**	24.1±4.0	23.7±3.6	0.259
Heart failure	39 (9.7%)	23 (16.2%)	16 (6.2%)	**0.002**	22 (12.0%)	17 (7.8%)	0.177
Hypertension	315 (78.6%)	119 (83.8%)	196 (75.7%)	0.075	162 (88.5%)	154 (70.6%)	**<0.001**
Diabetes	118 (29.4%)	47 (33.1%)	71 (27.4%)	0.253	67 (36.6%)	51 (23.4%)	**0.004**
Previous stroke/TIA	66 (16.5%)	27 (19.0%)	39 (15.1%)	0.326	37 (20.2%)	29 (13.3%)	0.078
Previous MI or PAD	44 (11.0%)	17 (12.0%)	27 (10.4%)	0.621	32 (17.5%)	12 (5.5%)	**<0.001**
CHA_2_DS_2_-VASc score	3.14±1.67	3.55±1.5	2.92±1.71	**<0.001**	3.86±1.47	2.40±1.51	**<0.001**
CACS	331.6±746.6	392.2±763.2	298.6±736.8	0.232	565.2±983.2	136.7±367.0	**<0.001**
Dyslipidemia	213 (53.1%)	71 (50.0%)	142 (54.8%)	0.403	99 (54.1%)	114 (52.3%)	0.763
Current or former smoker	171 (42.6%)	64 (45.1%)	107 (41.3%)	0.527	85 (46.4%)	86 (39.4%)	0.188
eGFR (mL/min/1.73 m^2^)	73.5±25.4	72.86±23.27	73.78±26.51	0.730	69.0±22.4	77.2±27.1	**0.001**
Echocardiography							
LA diameter (mm)	45.5±8.0	49.5±7.7	43.4±7.3	**<0.001**	46.7±8.2	44.6±7.7	**0.009**
LA volume index (mL/m^2^)	47.5±20.1	57.9±21.1	41.8±17.2	**<0.001**	49.9±20.6	45.5±19.5	**0.028**
LVEF (%)	62.9±9.4	61.0±10.7	63.9±8.4	**0.003**	63.4±9.3	62.5±9.4	0.342
Prior medications							
Antiplatelet	156 (38.9%)	56 (39.4%)	100 (38.6%)	0.915	69 (37.7%)	87 (39.9%)	0.682
Anticoagulant	104 (25.9%)	45 (31.7%)	59 (22.8%)	0.057	49 (26.8%)	55 (25.2%)	0.733
Statin	101 (25.2%)	40 (28.2%)	61 (23.6%)	0.337	49 (26.8%)	52 (23.9%)	0.564

Values are presented as mean ± standard deviation or as n (%). p-values < 0.05 are denoted by bold font.

^a^ CE risk factors: intracardiac thrombus, dense spontaneous echo contrast, or low left atrial appendage flow velocity (mean peak flow velocity ≤20 cm/s);

^b^ non-CE risk factors: intracranial arterial stenosis, significant carotid stenosis, or complex aortic plaque.

AF = atrial fibrillation; CACS = coronary artery calcium score; CE = cardioembolic; eGFR = estimated glomerular filtration rate; LA = left atrium; LVEF = left ventricular ejection fraction; MI = myocardial infarction; NVAF = non-valvular atrial fibrillation; PAD = peripheral artery disease; RWMA = regional wall motion abnormality; TIA = transient ischemic attack.

On multivariate logistic regression analysis, persistent AF (odds ratio [OR] 2.77, 95% confidence interval [CI] 1.63–4.70, p<0.001), high BMI (OR 1.12 per 1 kg/m^2^, 95% CI 1.04–1.20, p = 0.003), history of heart failure (OR 3.01, 95% CI 1.47–7.04, p = 0.003), and large LA volume index (OR 1.03 per 1 mL/m^2^, 95% CI 1.02–1.05, p<0.001) were independently associated with the presence of CE risk factors. Old age (OR 1.06 per year, 95% CI 1.03–1.09, p<0.001), hypertension (OR 1.84, 95% CI 1.00–3.38, p = 0.049), diabetes (OR 1.73, 95% CI 1.05–2.85, p = 0.030), and high CACS (OR 1.10 per 100, 95% CI 1.04–1.16, p<0.001) were independently associated with the presence of non-CE risk factors (Table 2).

**Table 2:** Logistic regression analyses of the presence of CE or non-CE stroke risk factors in patients with non-valvular atrial fibrillation.

Predictors	At least one CE risk factor				At least one non-CE risk factor			
	Univariate		Multivariate		Univariate		Multivariate	
	OR (95% CI)	p-value	OR (95% CI)	p-value	OR (95% CI)	p-value	OR (95% CI)	p-value
Age (per year)	1.03 (1.01–1.05)	**0.015**	1.03 (0.99–1.06)	0.109	1.08 (1.05–1.10)	**<0.001**	1.06 (1.03–1.09)	**<0.001**
Sex (male)	1.04 (0.68–1.61)	0.849	1.42 (0.75–2.68)	0.283	1.15 (0.76–1.74)	0.501	0.88 (0.49–1.61)	0.685
Persistent AF	4.93 (3.15–7.70)	**<0.001**	2.77 (1.63–4.70)	**<0.001**	1.50 (1.01–2.23)	**0.043**	1.38 (0.83–2.30)	0.219
Body mass index (kg/m^2^)	1.07 (1.01–1.13)	**0.032**	1.12 (1.04–1.20)	**0.003**	1.03 (0.98–1.08)	0.301	1.06 (0.99–1.13)	0.062
Heart failure	2.94 (1.50–5.76)	**0.002**	3.01 (1.47–7.04)	**0.003**	2.07 (1.03–4.15)	**0.041**	1.36 (0.56–3.33)	0.496
Hypertension	1.63 (0.96–2.77)	0.071	1.04 (0.55–1.99)	0.900	3.36 (2.00–5.67)	**<0.001**	1.84 (1.00–3.38)	**0.049**
Diabetes	1.31 (0.84–2.04)	0.233	1.15 (0.68–1.95)	0.606	1.88 (1.21–2.92)	**0.005**	1.73 (1.05–2.85)	**0.030**
Previous stroke/TIA	1.32 (0.77–2.27)	0.308	1.49 (0.79–2.81)	0.219	1.60 (0.94–2.75)	0.085	1.33 (0.72–2.46)	0.364
Previous MI	0.65 (0.20–2.09)	0.473	0.33 (0.08–1.37)	0.125	2.46 (0.83–7.34)	0.106	1.71 (0.45–6.53)	0.431
CACS (per 100)	1.02 (0.99–1.04)	0.236	1.01 (0.97–1.04)	0.773	1.14 (1.08–1.21)	**<0.001**	1.10 (1.04–1.16)	**<0.001**
Dyslipidemia	0.82 (0.55–1.24)	0.355	0.73 (0.45–1.20)	0.213	1.11 (0.75–1.65)	0.597	0.94 (0.60–1.50)	0.808
Current or former smoker	1.17 (0.77–1.76)	0.467	1.09 (0.60–1.98)	0.790	1.28 (0.86–1.90)	0.225	1.66 (0.94–2.92)	0.081
eGFR (mL/min/1.73 m^2^)	1.00 (0.99–1.01)	0.729	1.00 (0.99–1.01)	0.696	0.99 (0.98–0.99)	**0.001**	1.00 (0.99–1.01)	0.387
LA volume index (mL/m^2^)	1.05 (1.03–1.06)	**<0.001**	1.03 (1.02–1.05)	**<0.001**	1.01 (1.00–1.02)	**0.036**	1.00 (0.99–1.01)	0.870
LVEF (%)	0.97 (0.95–0.99)	**0.003**	0.98 (0.95–1.01)	0.222	1.01 (0.99–1.03)	0.575	1.02 (0.99–1.06)	0.115

Age, sex and clinical variables that had statistical significance on univariate analysis were included in multivariate regression. p values <0.05 are denoted by bold font. CI = confidence interval; OR = odds ratio. Other abbreviations and definitions of CE and non-CE risk factors are presented in Table 1.

### The distribution of CE and non-CE risk factors depending on CHA_2_DS_2_-VASc scores

**Fig 2:**
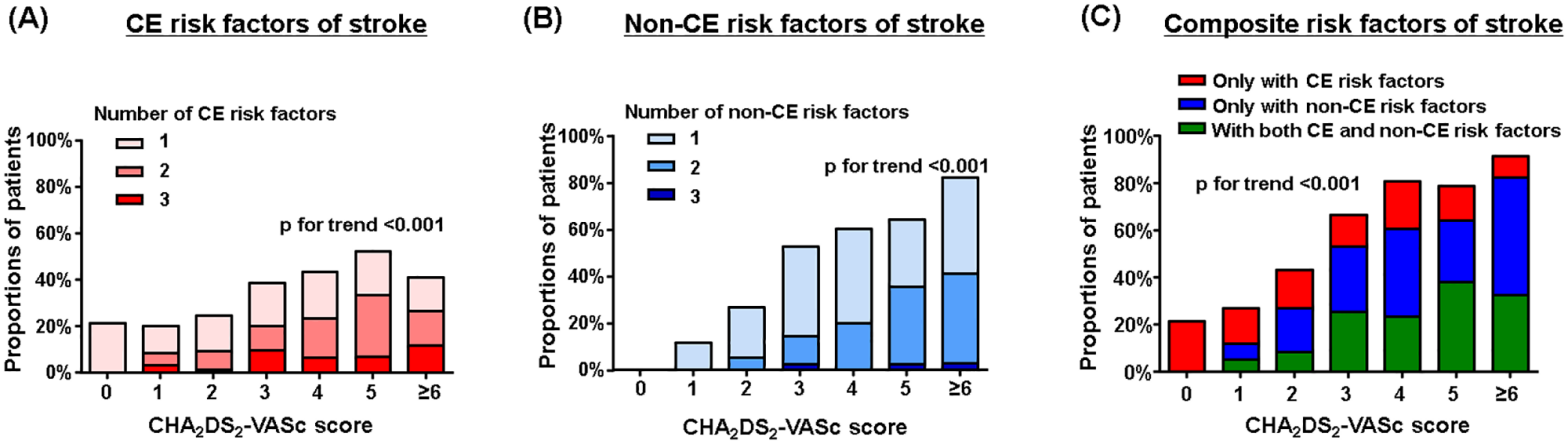
The burden of (A) cardioembolic (CE), (B) non-CE, and (C) composite risk factors of ischemic stroke according to CHA_2_DS_2_-VASc scores. CE risk factors: intracardiac thrombus, dense spontaneous echo contrast, or low left atrial appendage flow velocity (mean peak flow velocity ≤20 cm/sec). Non-CE risk factors: intracranial arterial stenosis, significant carotid stenosis, or complex aortic plaque. (Overall: n = 401; CHA_2_DS_2_-VASc score, 0: n = 14, 1: n = 60, 2: n = 74, 3: n = 83, 4: n = 94, 5: n = 42, ≥6: n = 34).

Table 3 shows the distribution of patients with CE and non-CE risk factors according to CHA_2_DS_2_-VASc scores. The prevalence of each risk factor significantly increased with increasing score and level of CHA_2_DS_2_-VASc risk category. In patients who were CHA_2_DS_2_-VASc 0 (n = 14) at the time of stroke, CE risk factors were found in 21.4% patients (3 of 14) and non-CE risk factors were not found. The numbers of CE, non-CE, and composite risk factors of ischemic stroke increased with increasing CHA_2_DS_2_-VASc scores (Fig 2).

**Table 3:** Distribution of patients and CE and non-CE risk factors of ischemic stroke according to CHA_2_DS_2_-VASc scores and risk categories.

	Patients	CE risk factors			Non-CE risk factors		
		Intracardiac thrombus	Dense SEC	Low LAA flow velocity	Intracranial arterial stenosis	Significant carotid stenosis	Complex aortic plaque
**Total number**	401	43	96	101	130	22	99
**CHA**_**2**_**DS**_**2**_**-VASc scores**							
**0**	14	0 (0.0%)	1 (7.1%)	2 (14.3%)	0 (0.0%)	0 (0.0%)	0 (0.0%)
**1**	60	3 (5.0%)	8 (13.3%)	8 (13.3%)	4 (6.7%)	1 (1.7%)	2 (3.3%)
**2**	74	4 (5.4%)	11 (14.9%)	11 (14.9%)	14 (18.9%)	2 (2.7%)	8 (10.8%)
**3**	83	13 (15.7%)	20 (24.1%)	24 (28.9%)	29 (34.9%)	6 (7.2%)	23 (27.7%)
**4**	94	14 (14.9%)	29 (30.9%)	26 (27.7%)	40 (42.6%)	4 (4.3%)	32 (34.0%)
**5**	42	5 (11.9%)	15 (35.7%)	19 (45.2%)	19 (45.2%)	5 (11.9%)	19 (45.2%)
**≥6**	34	4 (11.8%)	12 (35.3%)	11 (32.4%)	24 (70.6%)	4 (11.8%)	15 (44.1%)
**p for trend**		**0.030**	**<0.001**	**<0.001**	**<0.001**	**0.007**	**<0.001**
**CHA**_**2**_**DS**_**2**_**-VASc risk categories**							
**Low**	14	0 (0.0%)	1 (7.1%)	2 (14.3%)	0 (0.0%)	0 (0.0%)	0 (0.0%)
**Intermediate**	60	3 (5.0%)	8 (13.3%)	8 (13.3%)	4 (6.7%)	1 (1.7%)	2 (3.3%)
**High**	327	38 (11.6%)	87 (26.6%)	91 (27.8%)	126 (38.5%)	21 (6.4%)	97 (29.7%)
**p for trend**		**0.044**	**0.008**	**0.017**	**<0.001**	0.087	**<0.001**

Values are presented as n (%). p values <0.05 are denoted by bold font. Definitions of CE and non-CE risk factors are presented in Table 1. CE = cardioembolic; LAA = left atrial appendage; SEC = spontaneous echo contrast.

### Coronary artery calcium score (CACS) and the prevalence of non-CE risk factors

**Fig 3:**
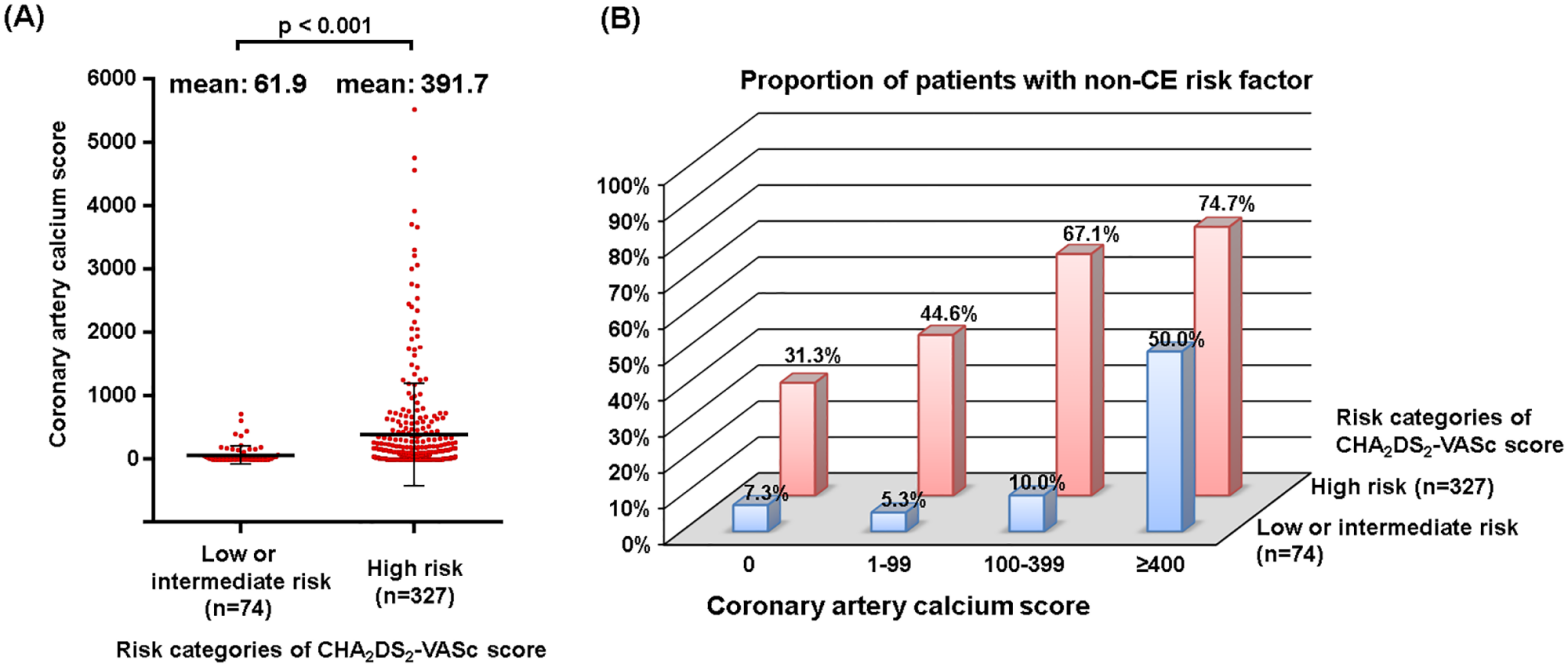
Coronary artery calcium scores and non-cardioembolic (non-CE) risk factors of ischemic stroke. (A) Comparison of coronary artery calcium scores between low/intermediate and high risk categories of CHA_2_DS_2_-VASc score. (B) Proportion of patients with non-cardioembolic (non-CE) risk factors according to risk categories of the CHA_2_DS_2_-VASc score and coronary artery calcium score.

Current guidelines include only ‘previous MI’ as one of the determinants of CHA_2_DS_2_-VASc scores, but the patients with high-risk CHA_2_DS_2_-VASc scores had higher CACS than patients with low/intermediate-risk CHA_2_DS_2_-VASc scores (391.7 ± 811.2 vs. 61.9 ± 138.8, p<0.001; Fig 3A). In the high risk category of the CHA_2_DS_2_-VASc score, the prevalence of non-CE risk factors (complex aortic plaque, significant carotid or intra-cranial arterial stenosis) increased with increasing CACS (p for trend <0.001, Fig 3B). Even in the low/intermediate risk category of CHA_2_DS_2_-VASc scores, this trend was consistent.

## Discussion

### Main findings

There are three major findings of this study. First, the prevalence and number of non-CE risk factors in patients with AF-associated ischemic stroke correlated with an increase in CHA_2_DS_2_-VASc scores. Second, in contrast to CE risk factors which were related to persistent AF, heart failure, overweight, and atrial enlargement, non-CE risk factors were associated with old age, hypertension, diabetes, and high CACS. Third, CACS had the added value in predicting non-CE risk factors of ischemic stroke regardless of the CHA_2_DS_2_-VASc risk category.

### Stroke mechanism in NVAF patients

When patients with new-onset ischemic stroke have NVAF, they are primarily classified as having CE stroke. Multiple factors, including AF burden [14], degree of atrial remodeling [14, 15], and hemodynamic loading [16], have been considered to increase the risk of CE stroke in NVAF. In this study, none of patients who experienced stroke at CHA_2_DS_2_-VASc 0 had non-CE risk factors, but 21.4% (3 of 14) of patients with CHA_2_DS_2_-VASc score 0 had CE risk factors. However, the end-points of previous studies on AF patients were any strokes, not just CE strokes. In the Stroke Prevention in Atrial Fibrillation (SPAF) III study about high risk AF patients, 24% of ischemic strokes were classified as non-CE stroke in NVAF patients with stroke despite adequate anti-coagulation [17]. Because most components of the CHA_2_DS_2_-VASc score consist of well-known atherosclerotic risk factors, atherosclerotic non-CE stroke can be increased in patients with higher CHA_2_DS_2_-VASc score. It is well known that patients with high CHA_2_DS_2_-VASc score have a high stroke risk even without AF [18, 19], Our results also shown that non-CE risk factors were found frequently in high risk NVAF patients (53.8% in CHA_2_DS_2_-VASc score ≥2), as well as CE risk factors (38.8% in CHA_2_DS_2_-VASc score ≥2). Therefore, if the patient is evaluated considering only CE risk because of the presence of AF at the time of the stroke, the important part for the stroke prevention will be lost. Although it is difficult to classify the mechanisms of stroke in patients with high-risk NVAF using brain imaging alone, searching for atherosclerotic non-CE sources, metabolic factors of atherosclerosis, and concomitant vascular disease cannot be omitted. Another important stroke mechanism is genetic factors. AF is a heritable disease, and some of the common genetic loci associated with AF are known risk factors of ischemic stroke [20].

### Stroke prevention for high risk AF patients

Some patients with NVAF will remain at high risk for ischemic stroke despite taking an oral anticoagulant. Previous reports showed that most ischemic strokes occurring in NVAF patients taking anticoagulants were non-CE [17, 21]. Evans et al. found that the rate of recurrence of lacunar infarction, which is usually associated with non-CE stroke, was higher than the risk of CE stroke over a 2-year follow-up of patients with ischemic stroke and AF receiving warfarin [22]. Anticoagulants are not effective in many of the non CE risk factors. Therefore, for optimizing prevention and treatment, the differentiation between CE and non-CE risks in NVAF patients and anti-thrombotic strategies for NVAF patients with apparent atherosclerotic non-CE risk factors should be established. Guidelines recommend antiplatelet and statin therapy for patients with a high risk of non-CE stroke [23, 24]. However, combination of anticoagulation and antiplatelet therapies is not recommended because it consistently increase major bleeding events [25]. Although LAA occlusion (LAAO) procedure was known to reduce the CE-risk in patients with NVAF [26], local therapy LAAO may not be enough to prevent ischemic stroke in patients with a very high risk of stroke and multiple non-CE risk factors. Therefore, further studies should be completed for more individualized stroke prevention in patients with NVAF, especially in patients with recurrent stroke under anticoagulation. There were several experimental reports indicating non-vitamin K oral anticoagulant (NOAC) attenuates atherosclerosis [27–29].

### Coronary artery calcium score as a marker of non-CE risk factors in NVAF patients

Among coronary artery disease, only ‘previous MI’ was included as vascular disease, which is one of the determinants of CHA_2_DS_2_-VASc scores [2, 3]. CACS is a noninvasive marker for plaque burden that can predict MI in the general population, and may reflect the presence of systemic atherosclerotic disease [30]. Previous studies reported that CACS can predict stroke in the general population [13]. In this study, we found that increased CACS was independently associated with non-CE risk factors, such as complex aortic plaque, significant carotid or intracranical arterial stenosis, regardless of previous MI history among the patients with NVAF and ischemic stroke. Therefore, CACS, which is a non-invasive atherosclerotic parameter, potentially provide additional information for risk of stroke in patients with NVAF in addition to the current determinants of CHA_2_DS_2_-VASc scores.

### Limitations

Our study has some limitations. First, this study is subject to all of the limitations inherent to a retrospective analysis. Second, we only included a selective group of patients with NVAF and ischemic stroke who underwent all following examinations; TEE, cardiac CT, carotid duplex sonography, and brain imaging. This retrospective non-consecutive inclusion has inherent risk of selection bias. Therefore, the findings of our study cannot be generalized to the entire NVAF population with ischemic stroke. However, the CHA_2_DS_2_-VASc score distribution of non-consecutively enrolled AF patients with stroke in this study was similar to that of consecutively enrolled AF patients with stroke in the previously published Yonsei Stroke Registry data of the same institution (S1 Fig) [31]. Third, we investigated only a limited number of CE and non-CE risk factors, which may not be representative of the total burden of risk. Fourth, it is unclear whether non-CE risk factors in high risk NVAF patients are associated phenomenon or have causal result relationship with stroke event because it was difficult to classify the mechanisms of stroke, CE or non-CE, in many patients.

## Conclusions

In NVAF patients with ischemic stroke, a high CHA_2_DS_2_-VASc score is strongly associated with non-CE risk factors as well as CE risk factors. Our findings suggest that atherosclerotic non-CE risk factors should be considered as potential mechanisms of stroke in NVAF patients with high CHA_2_DS_2_-VASc scores. And, CACS can be a good noninvasive marker to assess the risk of non-CE stroke in patients with NVAF.

## Supporting information

S1 Fig Comparison of CHA_2_DS_2_-VASc score distributions between non-consecutive AF patients of this study (Orange bar) and consecutive AF patients of previously published YONSEI Stroke Registry data (Blue bar). (DOCX)
