# Supplementary material for: Non-cardioembolic risk factors in atrial fibrillation-associated ischemic stroke
Source: PLoS One. 2018 Jul 20;13(7):e0201062. doi: 10.1371/journal.pone.0201062 (PMC6054400; doi:10.1371/journal.pone.0201062)
Supplement: S1 Fig — (DOCX) [file pone.0201062.s001.docx]

**Supplemental materials**

**S1 fig.** Comparison of CHA_2_DS_2_-VASc score distributions between non-consecutive AF patients of this study (Orange bar) and consecutive AF patients of previously published YONSEI Stroke Registry data (Blue bar).[1]


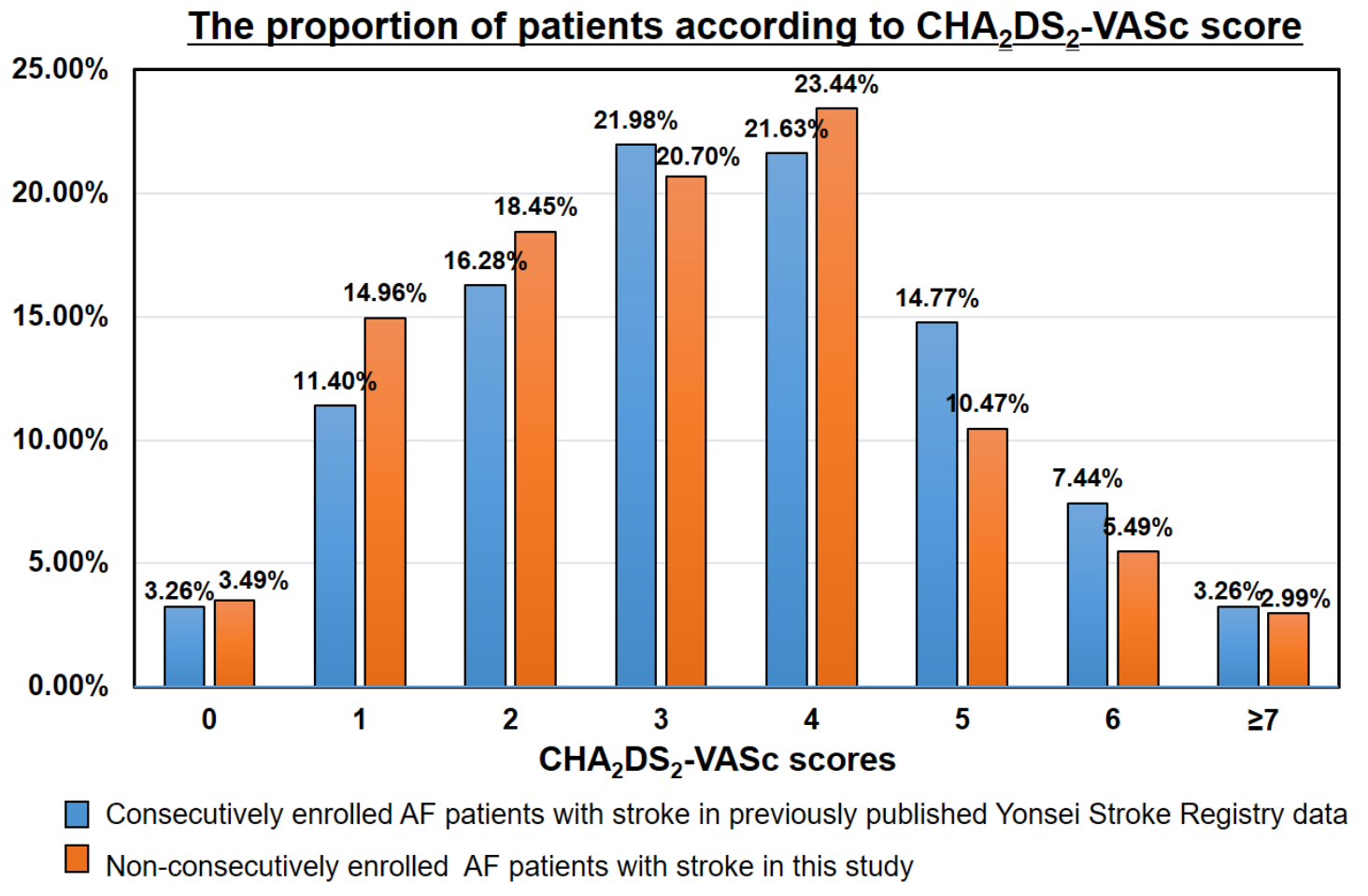


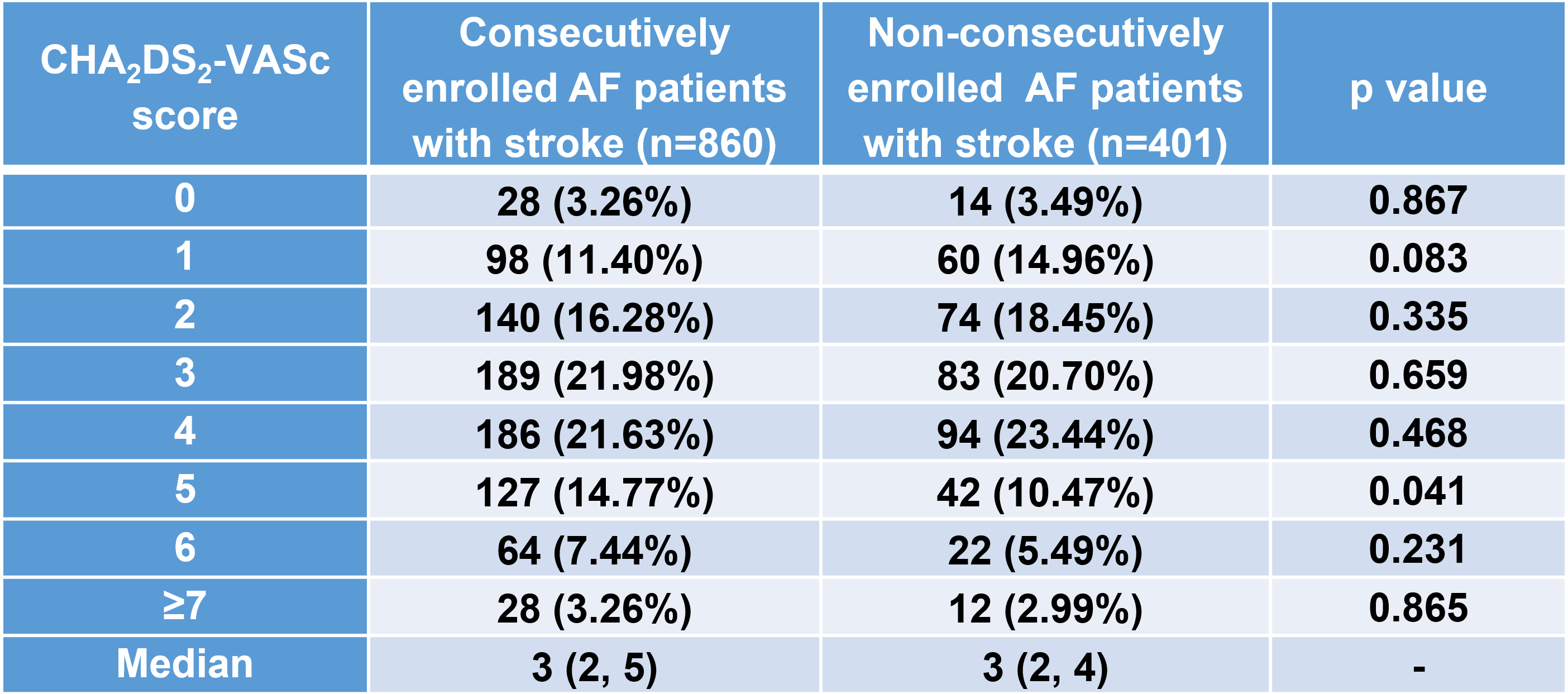


Values are presented as n (%) or as median (25th percentile, 75th percentile). Fisher’s exact test was used to determine the significance of differences between two groups. AF = atrial fibrillation.

**Reference**

1. Cha MJ, Kim YD, Nam HS, Kim J, Lee DH, Heo JH. Stroke mechanism in patients with non-valvular atrial fibrillation according to the CHADS2 and CHA2 DS2 -VASc scores. Eur J Neurol. 2012;19(3):473-479. Epub 2011/10/07. doi: 10.1111/j.1468-1331.2011.03547.x. PMID: 21972975.
